# Supplementary material for: Assessing the Ecotoxicity of Eight Widely Used Antibiotics on River Microbial Communities
Source: Int J Mol Sci. 2023 Nov 30;24(23):16960. doi: 10.3390/ijms242316960 (PMC10707202; doi:10.3390/ijms242316960)
Supplement: Supplementary file 1 [file ijms-24-16960-s001.zip › ijms-2699414-supplementary.pdf]

Table S1 Supplementary information

PARAMETER	UNITS	RESULT
Oxidability	mg/L	1.6
Suspended solids	mg/L	< 4 ± 20%
Dissolved solids	mg/L	1600 ± 20%
Calcium	mg/L Ca	144 ± 10%
Magnesium	mg/L Mg	9.7 ± 15%
Sodium	mg/L Na	278 ± 16%
Potassium	mg/L K	2.39 ± 15%
Ammonium	mg/L NH4	0.04
Alkalinity	mg/L CaCO3	640 ± 15%
Sulphates	mg/L SO4	29.8 ± 10%
Chlorides	mg/L Cl	61.5 ± 10%
Nitrates	mg/L NO3	< 2.29 ± 10%
Nitrites	mg/L NO2	1.44 ± 20%
Fluorides	mg/L F	< 0.5 ± 14%
Bromides	mg/L Br	< 0.20

Physicochemical analysis of the water samples (Gallego river, Zaragoza, Spain) from which the microbial communities under study were obtained.
